# Word-based characterization of promoters involved in human DNA repair pathways

**DOI:** 10.1186/1471-2164-10-S1-S18

**Published:** 2009-07-07

**Authors:** Jens Lichtenberg, Edwin Jacox, Joshua D Welch, Kyle Kurz, Xiaoyu Liang, Mary Qu Yang, Frank Drews, Klaus Ecker, Stephen S Lee, Laura Elnitski, Lonnie R Welch

**Affiliations:** 1 Bioinformatics Laboratory, School of Electrical Engineering and Computer Science, Ohio University, Athens, Ohio, USA; 2 Genomic Functional Analysis Section, National Human Genome Research Institute, National Institutes of Health, Rockville, Maryland, USA; 3 Department of Statistics, University of Idaho, Moscow, Idaho, USA; 4 Biomedical Engineering Program, Ohio University, Athens, Ohio, USA; 5 Molecular and Cellular Biology Program, Ohio University, Athens, Ohio, USA

## Abstract

**Background:**

DNA repair genes provide an important contribution towards the surveillance and repair of DNA damage. These genes produce a large network of interacting proteins whose mRNA expression is likely to be regulated by similar regulatory factors. Full characterization of promoters of DNA repair genes and the similarities among them will more fully elucidate the regulatory networks that activate or inhibit their expression. To address this goal, the authors introduce a technique to find regulatory genomic signatures, which represents a specific application of the genomic signature methodology to classify DNA sequences as putative functional elements *within *a single organism.

**Results:**

The effectiveness of the regulatory genomic signatures is demonstrated via analysis of promoter sequences for genes in DNA repair pathways of humans. The promoters are divided into two classes, the bidirectional promoters and the unidirectional promoters, and distinct genomic signatures are calculated for each class. The genomic signatures include statistically overrepresented words, word clusters, and co-occurring words. The robustness of this method is confirmed by the ability to identify sequences that exist as motifs in TRANSFAC and JASPAR databases, and in overlap with verified binding sites in this set of promoter regions.

**Conclusion:**

The word-based signatures are shown to be effective by finding occurrences of known regulatory sites. Moreover, the signatures of the bidirectional and unidirectional promoters of human DNA repair pathways are clearly distinct, exhibiting virtually no overlap. In addition to providing an effective characterization method for related DNA sequences, the signatures elucidate putative regulatory aspects of DNA repair pathways, which are notably under-characterized.

## Background

Genomic signature techniques were originally developed for identifying organism-specific characterizations [1,2]. Genomic signature methods carry the limitation that they were not designed for sub-categorization of sequences from within a single organism. To address this shortcoming, the authors present genomic signature techniques that can be used to identify regulatory signatures, i.e. to classify DNA sequences regarding related biological units *within *an organism, such as particular functions, pathways and tissues.

The term *genomic signature *was introduced by Karlin and Burge to refer to a function characterizing genomes based on compositional variation [2]. Karlin and others showed that a di-nucleotide odds-ratio was an effective genomic signature. In addition to the odds ratio, oligonucleotide frequencies (as n-mers) and machine learning methods have been employed to classify sequences based on their organism of origin [1,3-20], and to identify unique features of genomic data sets. Such approaches were effectively employed in a more refined focus examining tissue-specific categorization of regulatory sequences in liver or muscle [21-24].

Here, the authors employ a word-based genomic signature method. That is, given a group of related sequences, a set of characteristic subsequences is discovered. Each subsequence is called a *genomic word*. The set of characteristic subsequences and their attributes constitute a *word-based genomic signature*. It is hypothesized that each functionally related group of sequences has a detectable word-based signature, consisting of multiple genomic words. Furthermore, it is hypothesized that the genomic words that constitute a word-based genomic signature are functional genomic elements. Unlike most existing types of genomic signatures, a word-based genomic signature provides insights that are directly applicable to the problem of identifying functional DNA elements, because the words identify putative transcription factor binding sites.

The authors have identified two primary components of word-based genomic signatures that are useful for characterizing a set of related genomic sequences, *RGS*. The set of statistically overrepresented words that can be derived from RGS can be regarded as a word-based signature (*SIG1*) since it provides information about the complete set of potential control elements regulating the set of RGS. A second signature (*SIG2*) provides a set of words  related to the elements of  SIG1. The similarity between the sets can be measured based on evolutionary distance metrics, e.g. hamming and edit distance (also called Levenshtein distance, see Methods). In addition to SIG1 and SIG2 several post-processing steps built upon the two word-based signatures are undertaken to create the final regulatory genomic signature. These post-processing steps include *sequence clustering*, *co-occurrence analysis*, *biological significance analysis*, and a *conservation analysis*.

DNA repair genes represent a large network of genes that respond to DNA damage within a cell. Discrete pathways for DNA repair responses have been identified in the Reactome database [25]. A discernable feature among genes in these pathways is the promoter architecture. A large percentage of genes with DNA repair functions are regulated by bidirectional promoters [26,27], whereas the rest are regulated by unidirectional promoters. Bidirectional promoters fall between the DNA repair gene and a partner gene that is transcribed in the opposite direction. The close proximity of the 5' ends of this pair of genes facilitates the initiation of transcription of both genes, creating two transcription forks that advance in opposite directions. DNA repair genes rarely share bidirectional promoters with other DNA repair genes. Rather, they are paired with genes of diverse functions [26].

The formal definition of a bidirectional promoter requires that the initiation sites of the genes are spaced no more than 1000 bp from one another. Using these criteria the authors have comprehensively annotated the human and mouse genomes for the presence of bidirectional promoters, using *in silico *approaches [26,28]. Bidirectional promoters utilized repeatedly in the genome are known to regulate genes of a specific function [26] and serve as prototypes for complete promoter sequences for computational studies- i.e., one can deduce the full intergenic region because exons flank each side. These promoters represent a class of regulatory elements with a common architecture, suggesting a common regulatory mechanism could be employed among them. Recent molecular studies confirm that RNA PolII can dock at promoters while simultaneously facing both directions [29], rather than being restricted to a single direction.

DNA repair genes are likely to play a universal role in damage repair, therefore mutations that affect their regulation will become important diagnostic indicators in disease discovery. The authors have previously shown that bidirectional promoters regulate genes with characterized roles in both DNA repair and ovarian cancer [28]. A more detailed analysis of the regulatory motifs within this subset of promoters will address regulatory mechanisms controlling transcription of this important set of genes. This paper presents word-based genomic regulatory signatures based on statistically overrepresented oligonucleotides (6-8 mers) found in unidirectional and bidirectional promoters of genes in DNA repair pathways. The results demonstrate the effectiveness of using signatures for classifying biologically related DNA sequences. The oligonucleotides that comprise the signatures match known binding motifs from TRANSFAC [30] or JASPAR [31] databases. Furthermore, some examples overlap and agree with experimentally validated regulatory functions.

## Results

The effectiveness of genomic regulatory signatures that are based on **SIG1 **and **SIG2 **was addressed by analyzing promoter sequences for genes in DNA repair pathways of humans. The promoters were divided into two classes, the bidirectional promoters and the unidirectional promoters, and distinct genomic signatures were calculated for each class. The human DNA repair pathways included 32 bidirectional promoters and 42 unidirectional promoters. Bidirectional promoters had a GC content ranging between 47.55% and 77.09% with an average of 59.87% while unidirectional promoters varied from 38.00% to 68.09%, averaging 50.84%.

### Statistically overrepresented words

**Figure 1:**
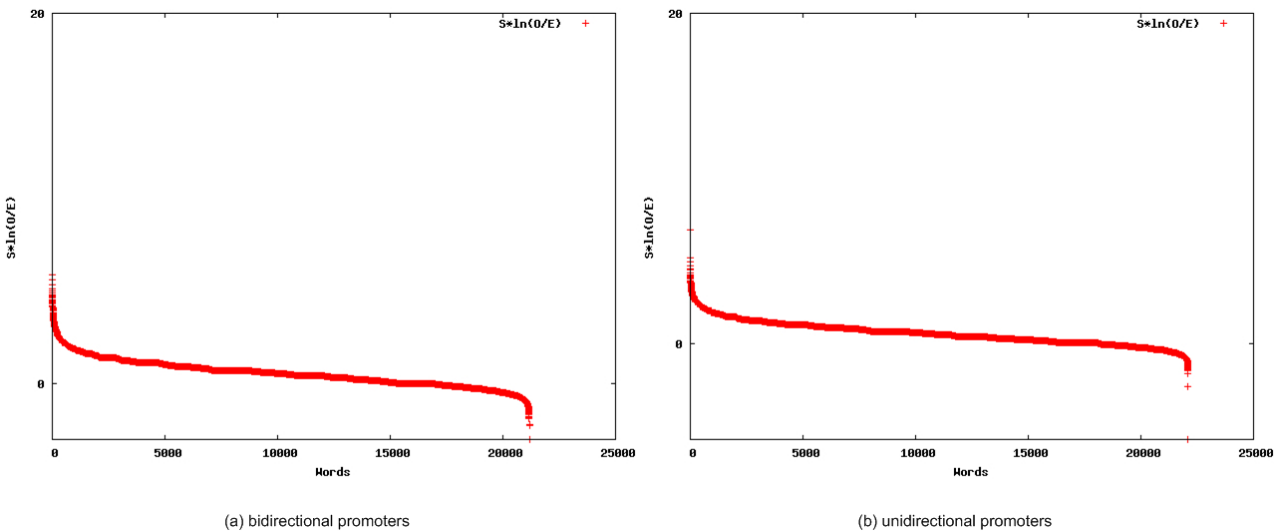
**Score-based scatterplots**. Shown here are the scatterplots for the scores of all words contained in the bidirectional promoter dataset (a) and the unidirectional promoter dataset (b) of the DNA repair pathways.

**Figure 2:**
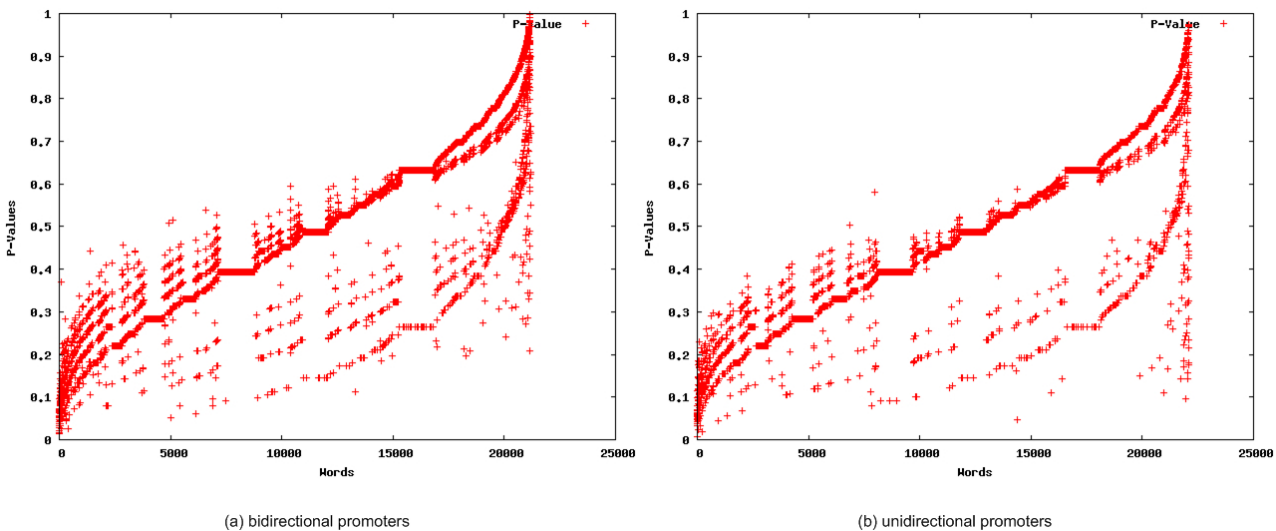
**P-Value-based scatterplots**. Scatterplots of the p-values for all words contained in the promoters of the DNA repair pathways exhibiting bi-directionality (a) and uni-directionality (b).

For each set of promoters, the statistically overrepresented words were identified. The top 25 overrepresented 8-mer words for each dataset are presented in Tables 1a and 1b, respectively (See Additional file 1 and Additional file 2 for the complete lists of words discovered in the bidirectional and unidirectional promoter set respectively). Each word is presented as an observed number or a statistical expectation, respectively, including the number of sequences the word is contained in (*S *or *E*_*S*_), the number of overall occurrences of the word (0 or *E*_*S*_), and a score measuring overrepresentation for the word . Additional information such as reverse complement words, their relative positions in the list of top words, palindromic words, and p-values assessing the statistical relevance of the appearance of the word are also presented. A comparison of Tables 1a and 1b reveals that the characteristic words for the two sets are distinct, with no overlaps. The significance of the selected 25 words can be seen by comparing their scores and p-values to the scores and p-values for all words, which are plotted in Figures 1 and 2).

**Table 1:** Top 25 words. The top 25 words for the bidirectional promoter set (a) and the unidirectional promoter set (b) of DNA-repair pathways. The words are sorted in descending order according to their statistical overrepresentation.

(a) Bidirectional									
Word	S	E_S_	O	E_O_	Sln(S/E_S_)	RevComp	Position	Palindrome	*P*-Value

TCGCGCCA	4	0.918299	4	0.9375	5.88611	TGGCGCGA	12538	No	0.015391

TCCCGGGA	8	3.97165	8	4.26667	5.60208	TCCCGGGA	2	Yes	0.068606

GGCCCGCC	10	5.85012	11	6.5	5.36123	GGCGGGCC	21073	No	0.066821

TCCCGGCT	6	2.54354	6	2.66667	5.14921	AGCCGGGA	NA	No	0.054084

CAGGGGCC	4	1.1085	4	1.13514	5.13315	GGCCCCTG	14546	No	0.028413

AGGGCCGT	5	1.80245	5	1.86667	5.10145	ACGGCCCT	613	No	0.04142

TCTGAGGA	5	1.84222	6	1.90909	4.99234	TCCTCAGA	5391	No	0.013499

CGTGGGGG	5	1.86693	5	1.93548	4.92572	CCCCCACG	20402	No	0.047015

TGCTGAGA	4	1.17067	4	1.2	4.91487	TCTCAGCA	NA	No	0.033766

CGCGGCCG	4	1.17067	4	1.2	4.91487	CGGCCGCG	20259	No	0.033766

TCTGGGAT	2	0.180188	2	0.181818	4.8138	ATCCCAGA	2854	No	0.014655

GGGGCCGG	5	1.92725	5	2	4.76672	CCGGCCCC	20866	No	0.052648

AGGGAGGG	6	2.73111	6	2.87234	4.7223	CCCTCCCT	9852	No	0.07159

AGAAAAGA	3	0.632564	3	0.642857	4.66976	TCTTTTCT	NA	No	0.027559

CGACTCCG	3	0.632564	3	0.642857	4.66976	CGGAGTCG	NA	No	0.027559

GGGCCAGG	7	3.61284	7	3.85714	4.6299	CCTGGCCC	19875	No	0.096315

ACTCCAGC	5	2.02051	5	2.1	4.53045	GCTGGAGT	NA	No	0.062121

CGGGCCGA	5	2.05153	5	2.13333	4.45426	TCGGCCCG	6128	No	0.065478

TGCGGAAT	2	0.220092	2	0.222222	4.41371	ATTCCGCA	NA	No	0.021321

GCCCCTCC	8	4.63031	9	5.03226	4.37454	GGAGGGGC	7041	No	0.070206

GCCGGCGA	3	0.707627	3	0.72	4.33335	TCGCCGGC	20143	No	0.036618

TGAAGCCA	4	1.38876	4	1.42857	4.23154	TGGCTTCA	NA	No	0.056996

GGCAGGGA	6	3.01111	6	3.18182	4.1367	TCCCTGCC	10531	No	0.103337

TGCCCGCG	5	2.19845	5	2.29167	4.10844	CGCGGGCA	NA	No	0.082773

CAGCAGCC	6	3.02748	6	3.2	4.10418	GGCTGCTG	19198	No	0.105399

(b) Unidirectional									

Word	S	E_S_	O	E_O_	Sln(S/E_S_)	RevComp	Position	Palindrome	*P*-Value

ACCCGCCT	4	0.716577	4	0.727273	6.87826	AGGCGGGT	19440	No	0.006562

CTTCTTTC	5	1.7686	5	1.81818	5.19624	GAAAGAAG	13567	No	0.037733

AGGAAACA	4	1.16659	4	1.19048	4.92885	TGTTTCCT	21667	No	0.032947

GCAGGGCG	6	2.75716	6	2.86957	4.66535	CGCCCTGC	1311	No	0.071337

GGGGCTGC	5	2.036	5	2.1	4.49226	GCAGCCCC	16359	No	0.062122

TCTTCTTC	4	1.30438	4	1.33333	4.48225	GAAGAAGA	NA	No	0.046491

GGGGAGTA	3	0.682407	3	0.692308	4.44222	TACTCCCC	17991	No	0.033211

ATTAAAAT	4	1.36853	4	1.4	4.29023	ATTTTAAT	16078	No	0.053723

CGGAAACC	3	0.750393	3	0.761905	4.15731	GGTTTCCG	NA	No	0.042101

TGGGCGGA	4	1.44679	4	1.48148	4.06778	TCCGCCCA	NA	No	0.063337

CGGCGGCG	3	0.787559	3	0.8	4.01229	CGCCGCCG	22091	No	0.047421

TTTTTTGA	3	0.787559	3	0.8	4.01229	TCAAAAAA	NA	No	0.047421

TTTCTCCA	4	1.48541	4	1.52174	3.96242	TGGAGAAA	2378	No	0.068398

AGCCGGCT	3	0.805285	3	0.818182	3.94551	AGCCGGCT	14	Yes	0.050071

CCTCTTTA	2	0.282982	2	0.285714	3.91104	TAAAGAGG	NA	No	0.033814

CGCCCCTT	6	3.12976	6	3.27273	3.90482	AAGGGGCG	21917	No	0.113859

GCGCCGCG	5	2.33164	5	2.41379	3.81433	CGCGGCGC	15062	No	0.097601

ATTCCCAG	3	0.843245	3	0.857143	3.80733	CTGGGAAT	21297	No	0.055985

TCTCCCCT	4	1.56036	4	1.6	3.7655	AGGGGAGA	18183	No	0.07881

TCCGCCGG	3	0.855341	3	0.869565	3.7646	CCGGCGGA	NA	No	0.057938

CTCCCGCT	3	0.867789	3	0.882353	3.72126	AGCGGGAG	NA	No	0.059981

TGCGCCGA	2	0.316812	2	0.32	3.68519	TCGGCGCA	3202	No	0.041483

GGGCGCCC	4	1.59514	4	1.63636	3.67732	GGGCGCCC	23	Yes	0.083901

GTGCGTTT	3	0.884961	3	0.9	3.66247	AAACGCAC	NA	No	0.062855

TTGGTCTC	4	1.60537	4	1.64706	3.65176	GAGACCAA	NA	No	0.085429

### Missing words

**Figure 3:**
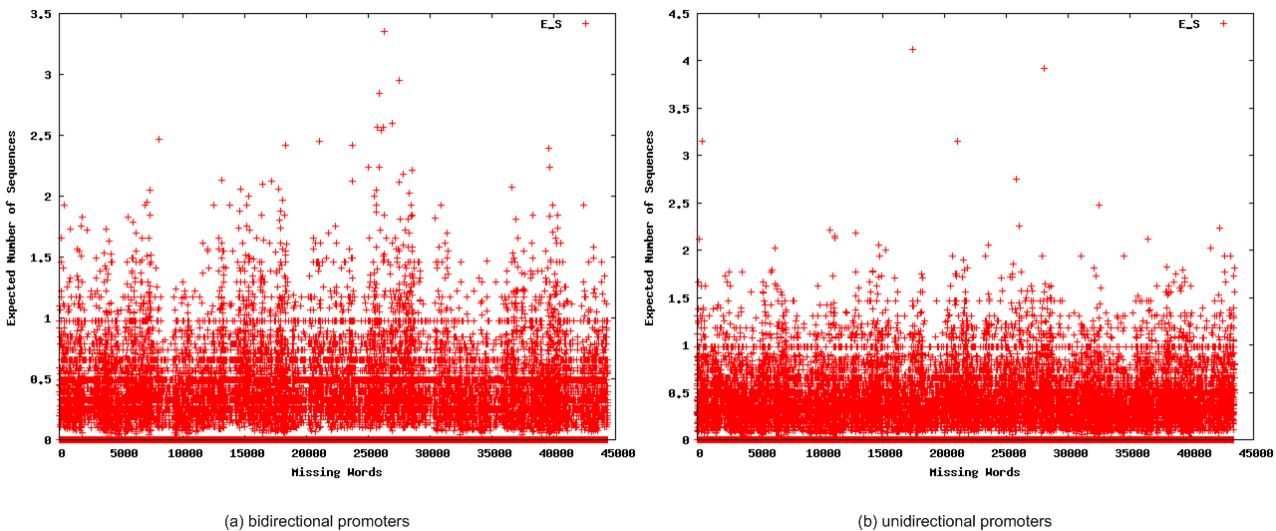
**Scatterplot of words not detected in the promoters**. Scatterplots for the expected number of sequence occurrences for every word not detected in the bidirectional (a) or unidirectional (b) promoters.

The dataset of bidirectional promoters and unidirectional promoters contained 21,076 and 22,101 unique words of length 8, respectively, out of 65,536 unique possibilities. Thus, in each set, more than 43,000 possible words did not occur (See Additional file 3 and Additional file 4 for the complete lists of non-occurring words). The missing words in each set were enumerated, and ranked in descending order by their E_S _values. The top 25 missing words are shown in Tables 2a and 2b. The scatterplot of the E_S _values for all missing words is shown in Figure 3; note the outlier values, which correspond with the words in Tables 2a and 2b. The utility of using missing words as regulatory signatures, as reported in the literature [32,33], was consistent with the observation of no overlapping words between bidirectional and unidirectional promoter sets.

**Table 2:** Top 25 words not part of promoter sets. The top 25 words that were *not *discovered as being part of the bidirectional (a) and unidirectional (b) promoter set of DNA-repair pathways. The words are sorted in descending order by the expected sequence occurrence (E_S_).

(a) Bidirectional		(b) Unidirectional	
Word	E_S_	Word	E_S_

GCGGCCCG	3.34859	CGCCCCTG	4.12035

GGAGGCGC	2.94738	GGCGGAGG	3.91749

GCCTCTCC	2.84694	AAAGGGGC	3.15484

GCTGAGGA	2.59894	CTGGTCTC	3.14943

GCCGGGGC	2.56699	GCCTGGGC	2.75165

GCGCCTCC	2.56699	GTTTGAAA	2.47933

GCGAGGCG	2.54354	GCGCGAGG	2.25604

AGTGGGGG	2.46473	TTCTTTTC	2.23192

CTGGAGGC	2.45191	ATTCTGGA	2.21123

CGGGGGTG	2.41485	CAGGCAGG	2.17759

GAGGGGAG	2.41485	ATTTTGTT	2.15141

TGCCCGCC	2.39066	CAAAAAAA	2.13045

GCACCCCC	2.23699	AAACCTCA	2.11329

GCCTCTGG	2.23699	TCCCGCCT	2.11329

TGCCTGCG	2.23699	CCCCGCCG	2.05605

GGGCTCGC	2.21328	GAGGAGGC	2.05268

GGCAGGGC	2.18091	AGCACTGG	2.02023

CAGCAAGG	2.1341	TTATCTGC	2.02023

CGAGGCCT	2.12325	CCGCCCCA	1.99873

GAGGGAAG	2.12325	CCCGCCCT	1.94132

GGAGCTGA	2.11348	CTCTTTCT	1.94132

CCTGTCCT	2.10187	GAGAGAGC	1.94132

TCCAGGAC	2.0706	GGCCCAAC	1.94132

CCAGGCCG	2.06039	GTCTGGGC	1.94132

CGCCTGTC	2.06039	TAGGGGGC	1.94132

### Word-based clusters

**Figure 4:**
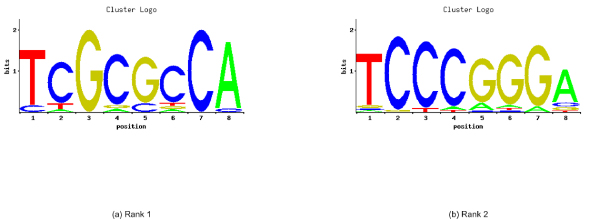
**Sequence logos for bidirectional promoters**. Sequence logos corresponding to the word-based clusters of the top 2 overrepresented words of the bidirectional promoters. Rank 1 (a) is corresponding to the word TCGCGCCA, while Rank 2 (b) refers to TCCCGGGA.

**Figure 5:**
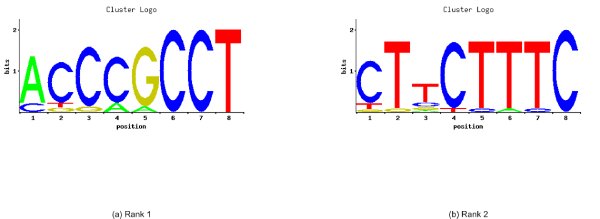
**Sequence logo for unidirectional promoters**. Sequence logos corresponding to the word-based clusters of the top 2 overrepresented words of the unidirectional promoters. Rank 1 (a) is corresponding to the word ACCCGCCT, while Rank 2 (b) refers to CTTCTTTC.

**Figure 6:**
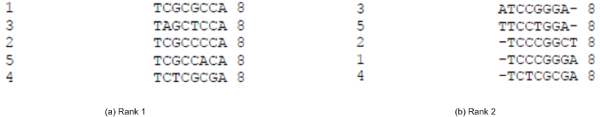
**Edit distance cluster for bidirectional promoters**. Sequence alignments corresponding to the word-based clusters of the top 2 overrepresented words of the bidirectional promoters. For each cluster, five words were chosen based on their overall overrepresentation in the promoter set. Rank 1 (a) is corresponding to the word TCGCGCCA, while Rank 2 (b) refers to TCCCGGGA.

**Figure 7:**
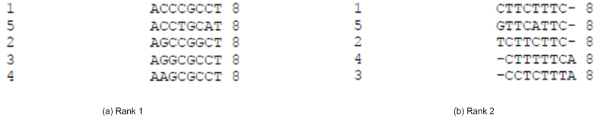
**Edit distance cluster for unidirectional promoters**. Sequence logos corresponding to the word-based clusters of the top 2 overrepresented words of the unidirectional promoters. Rank 1 (a) is corresponding to the word ACCCGCCT, while Rank 2 (b) refers to CTTCTTTC.

For the top 2 overrepresented words, clusters were created using two different distance metrics, hamming distance and edit distance (Tables 3, 4, 5, 6, See Additional File 5, 6, 7, 8 for the complete lists of hamming distance and edit distance based clusters for bidirectional and unidirectional promoters). Each table contains the set of words that clustered around a given 'seed' word. A comparison of the sequence logos for the hamming-distance-based clusters, presented in (Figures 4, 5), shows no overlap between the two promoter sets. Similarly, no overlap existed for clusters based on edit-distance (Figures 6, 7).

**Table 3:** Top 2 clusters for the bidirectional promoter. The word-based clusters for the two most overrepresented words for the bidirectional promoters. Rank 1 refers to word TCGCGCCA and Rank 2 to TCCCGGGA.

(a) Rank 1								
Word	S	E_S_	O	E_O_	Sln(S/E_S_)	RevComp.	Position	Palindrome

TCGCGCCA	4	0.918299	4	0.9375	5.88611	TGGCGCGA	12538	No

TCGCCCCA	3	0.805161	3	0.820513	3.94598	TGGGGCGA	2834	No

TAGCGCCA	1	0.263929	1	0.266667	1.33207	TGGCGCTA	4918	No

TCGAGCCA	1	0.469775	1	0.47619	0.755501	TGGCTCGA	NA	No

TCGCGACA	1	0.655751	1	0.666667	0.421975	TGTCGCGA	NA	No

TCGGGCCA	1	0.683955	1	0.695652	0.379863	TGGCCCGA	NA	No

TTGCGCCA	1	0.693903	2	0.705882	0.365423	TGGCGCAA	NA	No

TCGCGGCA	1	0.826074	1	0.842105	0.191071	TGCCGCGA	NA	No

TCGCGTCA	1	0.84063	1	0.857143	0.173604	TGACGCGA	4051	No

TCGCGCCC	1	1.51582	1	1.5625	-0.41596	GGGCGCGA	13089	No

CCGCGCCA	2	2.5054	2	2.625	-0.4506	TGGCGCGG	NA	No

(b) Rank 2								

Word	S	E_S_	O	E_O_	Sln(S/E_S_)	RevComp.	Position	Palindrome

TCCCGGGA	8	3.97165	8	4.26667	5.60208	TCCCGGGA	2	Yes

TCCAGGGA	2	0.941495	2	0.961538	1.50687	TCCCTGGA	NA	No

TCCCGAGA	2	1.05556	2	1.08	1.27816	TCTCGGGA	13248	No

TGCCGGGA	1	0.514348	1	0.521739	0.664856	TCCCGGCA	NA	No

TCCCGTGA	1	0.702073	1	0.714286	0.353718	TCACGGGA	NA	No

TCCCAGGA	4	3.71413	5	3.97222	0.296597	TCCTGGGA	19059	No

TCTCGGGA	2	1.73986	2	1.8	0.278683	TCCCGAGA	3074	No

ACCCGGGA	1	0.785281	1	0.8	0.241714	TCCCGGGT	20941	No

TCCCCGGA	1	0.852649	1	0.869565	0.159407	TCCGGGGA	NA	No

TCCCGCGA	1	1.01424	1	1.03704	-0.01414	TCGCGGGA	NA	No

TCCCGGAA	3	3.29619	3	3.5	-0.28247	TTCCGGGA	NA	No

TCCTGGGA	1	1.32696	1	1.36364	-0.28289	TCCCAGGA	13129	No

TCCCGGGG	3	3.34568	3	3.55556	-0.32717	CCCCGGGA	21071	No

TCCCGGGT	1	2.38044	1	2.48889	-0.86729	ACCCGGGA	13746	No

CCCCGGGA	1	2.78651	1	2.93333	-1.02479	TCCCGGGG	19211	No

GCCCGGGA	1	3.73853	2	4	-1.31869	TCCCGGGC	21163	No

TCCCGGGC	3	5.1829	4	5.68889	-1.64025	GCCCGGGA	21138	No

**Table 4:** Top 2 clusters for the unidirectional promoter. The word-based clusters for the two most overrepresented words for the bidirectional promoters. Rank 1 refers to word ACCCGCCT and Rank 2 to CTTCTTTC.

(a) Rank 1								
Word	S	E_S_	O	E_O_	Sln(S/E_S_)	RevComp.	Position	Palindrome

ACCCGCCT	4	0.716577	4	0.727273	6.87826	AGGCGGGT	19440	No

ATCCGCCT	1	0.132296	1	0.133333	2.02271	AGGCGGAT	NA	No

ACCAGCCT	2	0.738772	2	0.75	1.99183	AGGCTGGT	1303	No

AGCCGCCT	1	0.657331	1	0.666667	0.419567	AGGCGGCT	1056	No

ACCCACCT	1	0.738772	1	0.75	0.302766	AGGTGGGT	NA	No

ACGCGCCT	1	1.16147	1	1.18519	-0.14969	AGGCGCGT	NA	No

CCCCGCCT	1	2.45503	2	2.54545	-0.89814	AGGCGGGG	21912	No

(b) Rank 2								

Word	S	E_S_	O	E_O_	Sln(S/E_S_)	RevComp.	Position	Palindrome

CTTCTTTC	5	1.7686	5	1.81818	5.19624	GAAAGAAG	13567	No

CTACTTTC	1	0.180301	1	0.181818	1.71313	GAAAGTAG	NA	No

CTTCTTCC	1	0.304671	1	0.307692	1.18852	GGAAGAAG	5306	No

CTGCTTTC	2	1.15305	2	1.17647	1.10147	GAAAGCAG	9703	No

CGTCTTTC	1	0.371023	1	0.375	0.991491	GAAAGACG	20167	No

CTCCTTTC	3	2.36561	3	2.45	0.712729	GAAAGGAG	11346	No

CTTCTATC	1	0.607134	1	0.615385	0.499005	GATAGAAG	NA	No

CTTCCTTC	1	0.921427	1	0.9375	0.0818318	GAAGGAAG	10908	No

GTTCTTTC	1	1.07027	1	1.09091	-0.067912	GAAAGAAC	17502	No

CTTTTTTC	1	1.2055	1	1.23077	-0.186894	GAAAAAAG	NA	No

TTTCTTTC	2	3.4628	2	3.63636	-1.09786	GAAAGAAA	NA	No

**Table 5:** Edit cluster for bidirectional promoters. The word-based clusters for the two most overrepresented words for the bidirectional promoters according to the edit distance metric. Rank 1 refers to word TCGCGCCA and Rank 2 to TCCCGGGA.

(a) Rank 1								
Word	S	E_S_	O	E_O_	Sln(S/E_S_)	RevComp.	Position	Palindrome

TCGCGCCA	4	0.918299	4	0.9375	5.88611	TGGCGCGA	12538	No

TCGCCCCA	3	0.805161	3	0.820513	3.94598	TGGGGCGA	2834	No

TAGCTCCA	2	0.352982	2	0.357143	3.46897	TGGAGCTA	NA	No

TCTCGCGA	2	0.438673	2	0.444444	3.0343	TCGCGAGA	4937	No

TCGCCACA	2	0.455424	2	0.461538	2.95935	TGTGGCGA	4669	No

...								

(b) Rank 2								

Word	S	E_S_	O	E_O_	Sln(S/E_S_)	RevComp.	Position	Palindrome

TCCCGGGA	8	3.97165	8	4.26667	5.60208	TCCCGGGA	2	Yes

TCCCGGCT	6	2.54354	6	2.66667	5.14921	AGCCGGGA	NA	No

ATCCGGGA	2	0.395077	2	0.4	3.24364	TCCCGGAT	NA	No

TCTCGCGA	2	0.438673	2	0.444444	3.0343	TCGCGAGA	4937	No

TTCCTGGA	2	0.493082	2	0.5	2.80045	TCCAGGAA	9505	No

...								

**Table 6:** Edit cluster for unidirectional promoters. The word-based clusters for the two most overrepresented words for the unidirectional promoters according to the edit distance metric. Rank 1 refers to word ACCCGCCT and Rank 2 to CTTCTTTC.

(a) Rank 1								
Word	S	E_S_	O	E_O_	Sln(S/E_S_)	Rev.Comp.	Position	Palindrome

ACCCGCCT	4	0.716577	4	0.727273	6.87826	AGGCGGGT	19440	No

AGCCGGCT	3	0.805285	3	0.818182	3.94551	AGCCGGCT	14	Yes

AGGCGCCT	3	1.11427	3	1.13636	2.97124	AGGCGCCT	92	Yes

AAGCGCCT	4	2.15617	4	2.22727	2.47184	AGGCGCTT	5872	No

ACCTGCAT	2	0.592063	2	0.6	2.43458	ATGCAGGT	NA	No

...								

(b) Rank 2								

Word	S	E_S_	O	E_O_	Sln(S/E_S_)	Rev.Comp.	Position	Palindrome

CTTCTTTC	5	1.7686	5	1.81818	5.19624	GAAAGAAG	13567	No

TCTTCTTC	4	1.30438	4	1.33333	4.48225	GAAGAAGA	NA	No

CCTCTTTA	2	0.282982	2	0.285714	3.91104	TAAAGAGG	NA	No

CTTTTTCA	3	0.917377	3	0.933333	3.55455	TGAAAAAG	NA	No

GTTCATTC	2	0.359828	2	0.363636	3.43055	GAATGAAC	NA	No

...								

### Sequence-based clusters

**Figure 8:**
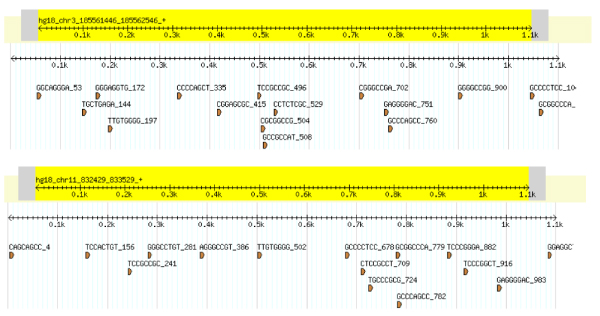
**GBrowse visualization for primary bidirectional sequence cluster**. The GBrowse visualization of the two sequences for the top sequence-based cluster in the bidirectional promoter set. Shown are the words from the set of top 60 words that are detected in these two sequences.

**Figure 9:**
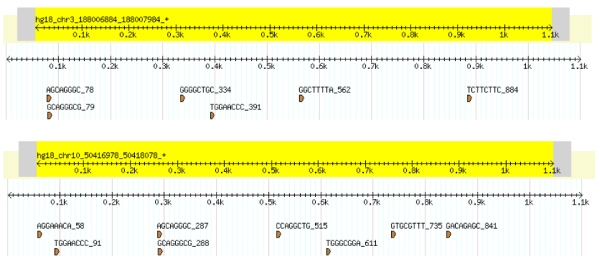
**GBrowse visualization for primary unidirectional sequence cluster**. The GBrowse visualization of the two sequences for the top sequence-based cluster in the unidirectional promoter set. Shown are the words from the set of top 60 words that are detected in these two sequences.

Sequences can be clustered and categorized into different families (and subfamilies). The sequence-based clusters presented here are restricted to two promoters per cluster. Sequence clustering is a measure of the co-existence of statistically overrepresented words shared between pairs of promoters as shown in Tables 7a,b. Each cluster contains IDs for the sequences that make up the cluster and the number of overrepresented words not shared within the cluster (distance). Sequences in each set were grouped into clusters based on the set of statistically overrepresented words. The shared words for the top-scoring sequence cluster of each data set were illustrated using the GBrowse environment [34] (Figures 8, 9). The visualization shows a strong positional correlation between the sequences of the top sequence cluster for the bidirectional promoters (Word: GCCCAGCC) and minor correlation between the sequences for the unidirectional promoters (Words: AGCAGGGC, GCAGGGCG).

**Table 7:** Sequence clusters (pairs of sequences). Sequence clusters containing pairs of sequences for the bidirectional (a) and unidirectional (b) promoter sets. Each sequence occurs in only one cluster. The sequences are clustered based on the number of words (within the top 60 overrepresented words) that are shared between them with the distance denoting the number of words not shared between them.

(a) Bidirectional			(b) Unidirectional		
Sequence 1	Sequence 2	Distance	Sequence 1	Sequence 2	Distance

chr3:185561446–185562546	chr11:832429–833529	54	chr10:50416978–50418078	chr3:188006884–188007984	57

chr19:53365272–53366372	chr19:7600339–7601439	55	chr12:52868924–52870024	chr7:73306574–73307674	57

chr11:18299718–18300818	chr15:41589928–41591028	56	chr5:68890824–68891924	chr19:55578407–55579507	58

chr4:57538069–57539168	chr19:48776246–48777346	56	chr6:30982955–30984055	chr9:99499360–99500460	58

chr11:107598052–107599152	chr12:131773918–131775018	56	chr10:131154509–131155609	chr19:50618917–50620017	58

chr13:107668425–107669525	chr1:11674165–11675265	57	chr5:86744492–86745592	chr17:30330654–30331754	58

chr6:43650922–43652022	chr16:2037768–2038868	57	chr11:118471287–118472387	chr8:55097461–55098561	58

chr22:36678663–36679763	chr11:61315725–61316825	58	chr16:13920523–13921623	chr8:101231014–101232114	58

chr5:60276548–60277648	chr22:40346240–40347340	58	chr5:131919528–131920628	chr19:1046236–1047336	58

chr11:93866588–93867688	chr3:130641442–130642542	58	chr12:108015528–108016628	chr16:56053079–56054179	59

chr17:7327421–7328521	chr17:1679094–1680194	58	chr1:28113723–28114823	chr2:216681376–216682476	59

chr20:5055168–5056268	chr15:38773660–38774760	58	chr8:91065972–91067072	chr4:39044247–39045347	59

chr14:19992129–19993229	chr11:66877493–66878593	59	chr14:60270222–60271322	chr11:47192088–47193188	59

chr17:38530557–38531657	chr13:31786616–31787716	59	chr7:7724663–7725763	chr11:62284590–62285690	59

chr12:122683333–122684433			chr13:33289233–33290333	chr12:116937892–116938992	59

chr5:82408167–82409267			chr9:109084364–109085464	chr7:101906286–101907386	59

chr2:127768122–127769222			chr8:42314186–42315286	chr19:50565569–50566669	59

chr12:102882746–102883846			chr3:9764704–9765804	chr14:49224583–49225683	59

			chr13:102295174–102296274	chr6:30790834–30791934	59

			chr12:912403–913503		

			chr2:128332074–128333174		

			chr7:44129555–44130655		

			chr11:73980276–73981376		

### Word co-occurrence

The promoter sets were characterized further by word co-occurrence analysis, in which word-pairs that appeared together more frequently than expected were identified. Interesting pairs of words were selected from the overrepresented words of Table 1 (Table 8a,b). Each word pair was characterized as the number of observed or expected occurrences for the word combination (*S *or *E*_*S*_) and a statistical overrepresentation score . No overlap was found between the bidirectional and the unidirectional set, nevertheless, the word pairs for the bidirectional promoter set achieved a higher number of sequence hits for the pairs.

**Table 8:** Word co-occurrence. The top 25 word pairs for the bidirectional (a) and unidirectional (b) promoter set. The word pairs are sorted in descending order by S*ln(S/E_S_) score.

(a) Bidirectional									
(a) Bidirectional					(b) Unidirectional				

Word 1	Word 2	S	E_S_	Sln(S/E_S_)	Word 1	Word 2	S	E_S_	Sln(S/E_S_)

TCTGAGGA	TCGCGCCA	3	0.0529	12.1158	GTTCATTC	TCCGCCGG	2	0.0073	11.2184

ACTCCAGC	TCGCGCCA	3	0.0580	11.8387	CTGTGTGC	TGCGCCGA	2	0.0074	11.1966

GCCCAGCC	TCCGCCGC	3	0.0722	11.1827	TGACGCGA	CTCCCGCT	2	0.0082	10.9997

GCCCAGCC	CGGAGCGC	2	0.0087	10.8711	AGCCGGCT	GGGGAGTA	2	0.0131	10.0590

TGCCCGCG	TCCCGGGA	4	0.2729	10.7404	ATTGCAGG	ATTCTCTC	2	0.0169	9.5459

GGCAGGGA	GGGCCAGG	4	0.3400	9.8609	GGGGAGTA	AGGAAACA	2	0.0190	9.3177

TCCCGGGA	TCGCGCCA	3	0.1140	9.8112	CTGGGAGC	GTTCATTC	2	0.0218	9.0337

AGCCTGTC	TCCCGGGA	3	0.1158	9.7646	CCTTCCGA	CTGGGAGC	2	0.0240	8.8439

GGAGGCTG	TCGCGCCA	3	0.1173	9.7250	TGGGCGGA	ACCCGCCT	2	0.0247	8.7895

TCCGCCGC	GCCCCTCC	4	0.3554	9.6830	TTTCTCCA	CGGAAACC	2	0.0265	8.6446

AGAAAAGA	TCGCGCCA	2	0.0182	9.4042	CCCCCGCG	ACCCGCCT	2	0.0280	8.5339

GCCCAGCC	GCCCCTCC	3	0.1360	9.2808	TCCGCCGG	GGGGCTGC	2	0.0415	7.7522

TGCCAAAA	GCCGGCGA	2	0.0195	9.2604	AGCTGGCT	CCAGGCTG	2	0.0422	7.7192

CAGCAGCC	TGCGGAAT	2	0.0208	9.1297	TTGGTCTC	AGGAAACA	2	0.0446	7.6068

AGGGCCGT	TCCCGGCT	3	0.1433	9.1249	CTGGGAGC	TCCGCCGG	2	0.0519	7.3020

CCTCCAGA	TTCCACCC	2	0.0216	9.0521	CTTTTCTC	GCGCCGCG	2	0.0545	7.2046

CGAGGAGA	TCGCGCCA	2	0.0220	9.0204	ATTGCAGG	ATTAAAAT	2	0.0585	7.0639

TCCGCCGC	CGGAGCGC	2	0.0228	8.9501	TGGAACCC	GCAGGGCG	2	0.0645	6.8693

ACCCTCGT	AGGGAGGG	2	0.0253	8.7380	GGGCAGGC	AGCTGGCT	2	0.0657	6.8326

GCCCAGCC	TCCACTGT	2	0.0254	8.7315	TTGGTCTC	CTTCTTTC	2	0.0676	6.7745

CAGCAGCC	AGGGCCGT	3	0.1705	8.6024	CTTTTTCA	CGCCCCTT	2	0.0684	6.7522

TGCCCGCG	TCCCGGCT	3	0.1747	8.5291	GCAGGGCG	AGGAAACA	2	0.0766	6.5251

CCCAGGAC	AGAGAGCT	2	0.0291	8.4590	GGGCAGGC	TTTCTCCA	2	0.0939	6.1181

TCTGGGAT	GGCCCGCC	2	0.0329	8.2123	CTGGGAGC	TCTCCCCT	2	0.0947	6.0996

AGCCGGGC	AGAAAAGA	2	0.0333	8.1930	AGCAGGGC	GGCTTTTA	2	0.0956	6.0805

### Comparison of word-based properties

**Figure 10:**
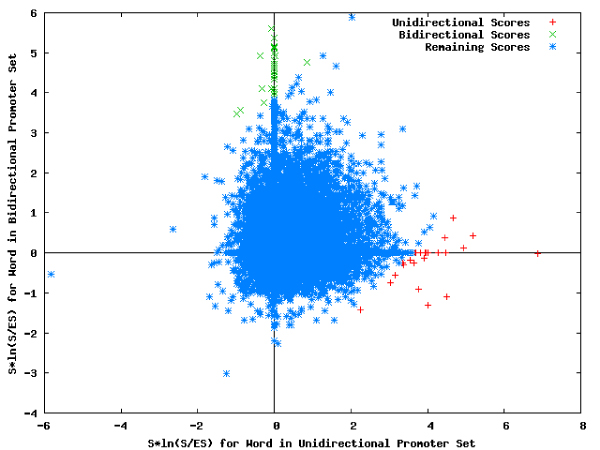
**Comparison analysis: plot for complete set of words**. Comparison of the words detected for the two promoter sets based on their computed overrepresentation scores.

**Figure 11:**
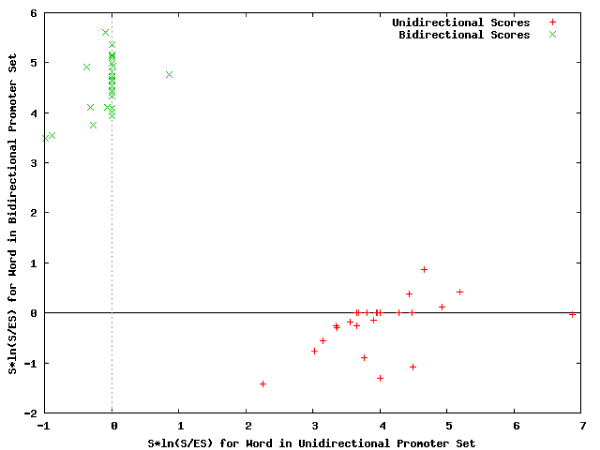
**Comparison analysis: plot for distinctive words**. The words descriptive of the unidirectional promoter set (red) and the bidirectional promoter set (green). Words that are not sufficiently descriptive of either data set are eliminated from the plot.

**Figure 12:**
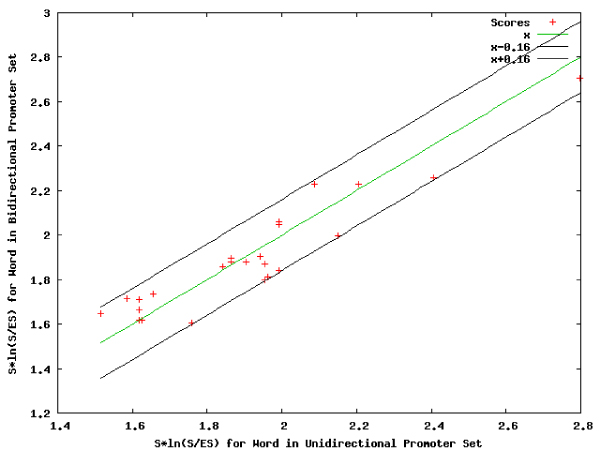
**Comparison analysis: plot for general words**. The words that are significantly correlated in both data sets.

The distances between the scores for different word sets (Figure 10) provided a basis for discriminating among bidirectional promoters and unidirectional promoters, (Table 9 and Figure 11), whereas similarities were identified from correlated words (Table 10 and Figure 12). These tables and figures show that word-based genomic regulatory signatures can be used to describe promoter sets based on their uniqueness.

**Table 9:** Unique and interesting words for the promoter sets. The words for the unidirectional and bidirectional promoter set which exhibit a significant score-based distance to the other data set.

(a) Unidirectional				(b) Bidirectional			
Word	Unidirectional	Bidirectional	Distance	Word	Unidirectional	Bidirectional	Distance

ACCCGCCT	6.87826	-0.0263597	4.882303411	TCCCGGGA	-0.0850495	5.60208	-4.021407835

GGGGCTGC	4.49226	-1.0872000	3.945274001	GGCCCGCC	0	5.36123	-3.790962089

CGGCGGCG	4.01229	-1.3139900	3.766248706	CGCGGCCG	-0.3641650	4.91487	-3.732841447

AGGAAACA	4.92885	0.1254760	3.396498328	TCCCGGCT	0	5.14921	-3.641041309

CTTCTTTC	5.19624	0.4219750	3.375915157	CAGGGGCC	0	5.13315	-3.629685174

TCCGCCGG	3.76460	-0.8986470	3.297413576	AGGGCCGT	0	5.10145	-3.607269889

TCTTCTTC	4.48225	0	3.169429370	TCTGAGGA	0	4.99234	-3.530117468

ATTAAAAT	4.29023	0	3.033650726	CGTGGGGG	0.0180292	4.92572	-3.470261445

GGGGAGTA	4.44222	0.3737000	2.876878081	TCTGGGAT	0	4.81380	-3.403870623

CGCCCCTT	3.90482	-0.1463740	2.864626749	AGGGAGGG	0	4.72230	-3.339170353

TTTTTTGA	4.01229	0	2.837117467	AGAAAAGA	0	4.66976	-3.302018963

TTTCTCCA	3.96242	0	2.801854052	GGGCCAGG	0	4.62990	-3.273833686

AGCCGGCT	3.94551	0	2.789896876	ACTCCAGC	0	4.53045	-3.203511917

TTGGTCTC	3.65176	-0.2608830	2.766656398	CCCCAGCT	-0.9904730	3.48143	-3.162112936

GCGCCGCG	3.81433	0	2.697138609	CGGGCCGA	0	4.45426	-3.149637451

ATTCCCAG	3.80733	0	2.692188861	TCCGCCGC	-0.8886350	3.55395	-3.141381979

GCAGGGCG	4.66535	0.8645290	2.687586303	TGCCCGCG	-0.3137370	4.10844	-3.126951344

GAGGGGCG	3.03108	-0.7557900	2.677721456	TGCGGAAT	0	4.41371	-3.120964271

CCCCCGCG	3.55664	-0.1908410	2.649869227	GCCGGCGA	0	4.33335	-3.064141170

AGGGGAGC	3.15866	-0.5635770	2.632019024	CAGCAGCC	-0.0679120	4.10418	-2.950114545

TGCGCCGA	3.68519	0	2.605822839	CGAGGAGA	0	4.09415	-2.895001228

CCGCGCCC	2.25420	-1.4189300	2.597295131	CGCAGGCG	-0.2779570	3.74626	-2.845551130

GTGCGTTT	3.66247	0	2.589757373	TTCCACCC	0	4.02098	-2.843262225

CTGGGAGC	3.36673	-0.2940760	2.588580747	TCGCCCCA	0	3.94598	-2.790229216

TGCCTCCC	3.34992	-0.2629130	2.554658714	GGGGCCGG	0.8548330	4.76672	-2.766121825

**Table 10:** Descriptive words for both the unidirectional and bidirectional promoter sets. The top 25 words that are correlated in the two promoter sets, according to their overrepresentation scores. The Words had to be overrepresented according to SlnSES with at least a score of 1.5. Shown are the words with a distance between -0.11 and 0.11.

Word	Unidirectional	Bidirectional	Distance
CTTTGGCC	2.08857	2.23024	-0.100175818

AGGCAGGA	1.51526	1.64780	-0.093719933

CTCAGGAT	1.58527	1.71375	-0.090849079

GGGGGGAC	1.61803	1.70814	-0.063717392

CTTGCGGA	1.65530	1.73350	-0.055295750

CTGAGCAG	1.99183	2.05890	-0.047425652

GCCTGAGG	1.99183	2.04796	-0.039689904

TGAAGTGG	1.61803	1.66175	-0.030914708

GCCATCCG	1.86393	1.89589	-0.022599133

AGGTTGCA	2.20477	2.23024	-0.018010010

TCTGTGCC	1.84096	1.85915	-0.012862272

TACCACTA	1.86393	1.88037	-0.011624835

CAAAGAAT	1.61803	1.61872	-0.000487904

ACCGCTCA	1.61803	1.61872	-0.000487904

TATCTTAG	1.61803	1.61872	-0.000487904

AGAGTTCC	1.62605	1.61872	0.005183093

GTCGGCTT	1.90512	1.88037	0.017500893

CGCGCGCA	1.94164	1.90263	0.027584236

CAGGCCAG	1.95383	1.86972	0.059474751

ACAGAAAG	2.79686	2.70295	0.066404398

GTCAGGAG	2.40520	2.25776	0.104255824

GGAAGTGA	1.96108	1.81095	0.106157941

TAGAGAGC	1.99183	1.84125	0.106476139

TGCCAGGG	1.75813	1.60511	0.108201480

GCACAAGC	1.95383	1.80053	0.108399470

TTCACTTA	2.15055	1.99725	0.108399470

### Regulatory Database Lookup

We developed a method [35] to determine if these signatures matched any known motifs from TRANSFAC or JASPAR (Table 11). The words from bidirectional promoters matched known motifs in 8/10 cases, with the words from unidirectional promoters matching known motifs in 8/10 cases as well. Compared to the consensus sequences of the known motifs, the matches were off by no more than one letter. Some of the matches corresponded to nucleotide profiles determined from collections of phylogenetically conserved, cis-acting regulatory elements [36]. Imperfect matches resulted from bases that flanked the core motifs (Table 11a, b) (see also [37]). Such events decreased the detection score to slightly above the threshold of 85% similarity. Overall, the findings in Table 11 validate that the signatures have biological relevance and suggest that the remaining signatures, which do not match known motifs could represent novel binding sites.

**Table 11:** Lookup results for interesting words in the promoters. Information about the regulatory function of the top 10 overrepresented words for the bidirectional and unidirectional promoter set based on lookups in the TRANSFAC and JASPAR databases.

(a) Bidirectional					
Sequence	Transcription Factor (Matrix Id^a^)	Sequence (bottom) aligned to matrix consensus^b^	Matches^c^	Avg. Score^d^	Score Range^e^

TCGCGCCA	PF0112^f^	KTGGCGGGAA TGGCGCGA	4/6	89.0	86.5–96.8

TCCCGGGA	STAT5A	TTCYNRGAA TCCCGGGA	8/16	86.7	86.7–86.7

GGCCCGCC	SP1 (V$SP1_01)	DRGGCRKGSW GGCGGGCC	8/13	90.2	86.5–90.8

TCCCGGCT	ELK1 (MA0028)	NNNMCGGAAR AGCCGGGA	3/6	86.9	86.5–87.7

CAGGGGCC	V$WT1_Q6	SVCHCCBVC GGCCCCTG	5/6	87.4	85.0–91.1

AGGGCCGT	MYB (V$MYB_Q3)	NNNBNCMGTTN AGGGCCGT	2/7	91.2	89.8–92.6

TCTGAGGA	TFIIA (V$TFIIA_Q6)	TMTDHRAGGRVS TCTGAGGA	2/8	88.1	85.8–90.5

CGTGGGGG	E2F (V$E2F1_Q3)	BKTSSCGS CGTGGGGG	6/6	87.3	87.3–87.3

TGCTGAGA	No match.				

CGCGGCCG	No match.				

(b) Unidirectional					

Sequence	Transcription Factor (Matrix Id^a^)	Sequence (bottom) aligned to matrix consensus^b^	Matches^c^	Avg. Score^d^	Score Range^e^

ACCCGCCT	SP1 (V$SP1_01)	DRGGCRKGSW AGGCGGGT	4/7	86.2	85.9–87.3

CTTCTTTC	No match.				

AGGAAACA	NFAT (V$NFAT_Q4_01)	NWGGAAANWB AGGAAACA	5/5	87.3	85.8–88.1

GCAGGGCG	PF0096^f^	YGCANTGCR GCAGGGCG	10/10	86.8	86.5–87.1

GGGGCTGC	LRF (V$LRF_Q2)	VDVRMCCCC GCAGCCCC	5/8	85.4	85.4–85.4

TCTTCTTC	No match.				

GGGGAGTA	FOXC1 (MA0032)	NNNVNGTA GGGGAGTA	4/4	95.5	95.5–95.5

ATTAAAAT	OCT1 ($OCT1_06)	MWNMWTKWSATRYN ATTTTAAT	4/9	86.9	86.5–87.5

CGGAAACC	AREB6 (V$AREB6_04)	VBGTTTSNN GGTTTCCG	3/3	92.2	88.3–95.8

TGGGCGGA	GC (V$GC_01)	NNDGGGYGGRGYBD TGGGCGGA	4/5	90.3	85.1–95.2

a. JASPAR id or TRANSFAC id.

b. The consensus is in IUPAC notation: R = G or A, Y = T or C, M = A or C, H = not G, K = G or T, W = A or T, B = not A, S = G or C, V = not T, N = anything.

c. Number of occurrences of the matrix that scored greater than 85% in the dataset.

d. Average score for the occurrences meeting the 85% threshold.

e. Range of scores for the occurrences meeting the 85% threshold.

f. A profile that was extracted from phylogenetically conserved gene upstream elements.

### Conservation analysis

To address selective constraint in the word sets, sequence conservation was examined for pairs of co-occurring words. The top ten word-pairs from the unidirectional and bidirectional datasets were examined in 28-way sequence alignments using the PhastCons [38] dataset in the UCSC Human Genome Browser [39]. The results are presented in Table 12. The bidirectional promoters revealed 9/10 word sets had a record of sequence conservation in one or both words (Table 12a). The analysis of the unidirectional promoters, presented in Table 12b, showed partial conservation in only one of the word-pairs.

**Table 12:** Conservation analysis. The results for conservation analysis of the top 10 word pairs in the bidirectional (a) and unidirectional (b) promoter set. For each word pair, the occurrence location of the pair is given, as well as an identifier for the conservation of the sites, and a PhastCons score for the quality of the conservation across 28 organisms. Conservation can be categorized as: none (no word was conserved), partial (one word was conserved) and complete (all words were conserved).

(a) Bidirectional					
Word 1	Word 2	Location	Conservation	Hit	Score

TCTGAGGA	TCGCGCCA	chr19:53365272–53366372	None		

		chr19:48776246–48777346	None		

		chr19:7600339–7601439	Partial	TCGCGCCA	385

ACTCCAGC	TCGCGCCA	chr4:57538069–57539168	None		

		chr19:48776246–48777346	None		

		chr19:7600339–7601439	Partial	TCGCGCCA	385

GCCCAGCC	TCCGCCGC	chr3:185561446–185562546	Partial	TCCGCCGC	310

		chr14:19992129–19993229	None		

		chr11:832429–833529	None		

GCCCAGCC	CGGAGCGC	chr3:185561446–185562546	None		

		chr14:19992129–19993229	None		

TGCCCGCG	TCCCGGGA	chr19:53365272–53366372	Partial	TCCCGGGA	390

		chr13:107668425–107669525	None		

		chr20:5055168–5056268	None		

		chr11:832429–833529	None		

GGCAGGGA	GGGCCAGG	chr19:53365272–53366372	Partial	GGGCCAGG	390

		chr22:40346240–40347340	Complete	GGCAGGGA	325

				GGGCCAGG	522

		chr5:60276548–60277648	None		

		chr12:131773918–131775018	None		

TCCCGGGA	TCGCGCCA	chr19:53365272–53366372	Partial	TCCCGGGA	390

		chr4:57538069–57539168	None		

		chr19:7600339–7601439	Partial	TCGCGCCA	385

AGCCTGTC	TCCCGGGA	chr17:38530557–38531657	None		

		chr13:107668425–107669525	Partial	AGCCTGTC	244

		chr4:57538069–57539168	None		

GGAGGCTG	TCGCGCCA	chr4:57538069–57539168	None		

		chr19:48776246–48777346	None		

		chr19:7600339–7601439	Partial	TCGCGCCA	385

TCCGCCGC	GCCCCTCC	chr3:185561446–185562546	Partial	TCCGCCGC	310

		chr14:19992129–19993229	None		

		chr1:11674165–11675265	Partial	GCCCCTCC	360

		chr11:832429–833529	None		

(b) Unidirectional					

Word 1	Word 2	Location	Conservation	Hit	Score

GTTCATTC	TCCGCCGG	chr7:73306574–73307674	None		

		chr12:52868924–52870024	Partial	TCCGCCGG	325

CTGTGTGC	TGCGCCGA	chr10:131154509–131155609	None		

		chr19:1046236–1047336	None		

TGACGCGA	CTCCCGCT	chr12:116937892–116938992	None		

		chr17:30330654–30331754	None		

AGCCGGCT	GGGGAGTA	chr6:30982955–30984055	None		

		chr16:13920523–13921623	None		

ATTGCAGG	ATTCTCTC	chr5:86744492–86745592	None		

		chr17:30330654–30331754	None		

GGGGAGTA	AGGAAACA	chr16:13920523–13921623	None		

		chr8:101231014–101232114	None		

CTGGGAGC	GTTCATTC	chr7:73306574–73307674	None		

		chr12:52868924–52870024	None		

CCTTCCGA	CTGGGAGC	chr5:68890824–68891924	None		

		chr7:73306574–73307674	None		

TGGGCGGA	ACCCGCCT	chr6:30982955–30984055	None		

		chr9:99499360–99500460	None		

TTTCTCCA	CGGAAACC	chr8:55097461–55098561	None		

		chr11:118471287–118472387	None		

### Biological implications

The words in the list of bidirectional promoters were examined for known biological evidence. For instance, the gene *POLH *has a known binding motif, TCCCGGGA, annotated as a *PAX-6* binding site in the cis-RED database . This is the same sequence as the second most common word in the bidirectional promoters. Along with sequences that cluster with this word, we found that 19/32 genes in the bidirectional promoter set had a match to this word cluster (cluster 2) within 1 kb of their TSS, while 15/32 bidirectional promoters had a match to the words of cluster 1. Furthermore, this word also represents a Stat5A recognition site (Table 11). The *RAD51 *gene, which is known to be regulated by *STAT5A*, showed two examples from this word cluster (TGCCGGGA and TCCCGGGC).

### Limitations of the approach

The presented approach does not attempt to automate the process of finding a small set of regulatory elements for a limited set of related genomic signatures like MEME [40] or AlignACE [41]. The different approach presented here produces more detailed information outside of the limited list by showing a larger (complete) set of words that are ranked based on their statistical significance. Additionally, word- and sequence-based clusters, word co-occurrences and functional significance of the words have been computed as a means of adding more detail to the retrieval of putative elements allowing a more informed interpretation of the actual regulatory function of a word.

## Conclusion

This paper presents a word-based genomic signature that characterizes a set of sequences with (1) statistically overrepresented words, (2) missing words, (3) word-based clusters, (4) sequence-based clusters and (5) co-occurring words. The word-based signatures of bidirectional and unidirectional promoters of human DNA repair pathways showed virtually no overlap, thereby demonstrating the signature's utility.

In addition to providing an effective characterization method for related DNA sequences, the signatures elucidate putative regulatory aspects of DNA repair pathways. Genes in DNA repair pathways contribute to diverse functions such as sensing DNA damage and transducing the signal, participating in DNA repair pathways, cell cycle signalling, and purine and pyrimidine metabolism. The synchronization of these functions implies co-regulatory relationships of the promoters of these genes to ensure the adequate production of all the necessary components in the pathway. We present a subtle, yet detectable signature for bidirectional promoters of DNA repair genes. The consensus patterns, detected as words and related clusters of words, provide a DNA pattern that is strongly represented in these promoters. Although the proteins that bind these sequences must be examined experimentally, the data show that a protein such as STAT5A could be involved in regulating many of these promoters. *STAT5A *has biological relevance in DNA repair pathways, playing a known role in the regulation of the *RAD51 *gene. We propose that this initial study of a network of DNA repair genes serve as a model for studies that examine regulatory networks. As the relationships among genes involved in DNA repair pathways are elucidated more thoroughly, the analyses of their regulatory relationships will gain more power to detect a larger number of DNA words that are shared in common among the network of genes. The results of this analysis are supported by evidence of sequence conservation and overlap between predicted sites and known functional elements.

## Methods

Two fundamental elements of word-based genomic signatures are created with the approach presented in [42,43]. SIG1 identifies the set of statistically overrepresented words, while SIG2 represents a set of words from SIG1 that is in itself similar to the elements of SIG1, based on a specific distance measure.

The set **SIG1 **is computed as described in [42,43], which is summarized as follows:

1. Identify maximally repeated words of length [m, n].

2. Remove low complexity words, redundant words, and words that are contained in repeat elements.

3. For each word compute a 'score' that characterizes the statistical overrepresentation of the word.

4. Select the words with the highest scores.

The set **SIG2 **is found by taking each of the elements of **SIG1 **and performing 'word clustering'. For each word *w *∈ **SIG1**, this involves a two-step process:

1. Construct a set (cluster) of words from **RGS **that have a 'distance' of no more than *h *from word *w*. Hamming distance and edit distance are used for this step.

2. Construct a *motif *that characterizes the set of words found in step 1.

### Word-based signature (SIG1)

As the foundation of the signature generation it is necessary to compute the set of distinct words *W*^*wc *^in a set of input sequences *S*. In order to determine the statistical significance of *w *∈ *W*^*wc *^it is necessary to count the total number of occurrences of a given word *w*_*j*_, *o*_*j*_, as well as the number of sequences containing the word, *s*_*j*_. The occurrence information is modelled as a set of tuples . Assuming a binomial model for the distribution of words across the input sequences, it is possible to model the total occurrence of a word *w *by introducing the random variable , where *l *is the complete sequence length, *v *the length of *w*, and *Y*_*i *_a binary random variable indicating if a word occurs at position *i*, or not, leading to the series of yes/no Bernoulli experiments. An expected value for the specific number of occurrences for a word *w *can then be computed as  where *p*_*w *_is the probability of word *w*. Following a similar modelling approach, the expected number of sequences a word occurs in is given by . The actual probabilities are determined by a homogenous Markov chain model of a specific order *m*. Based on the expected values we compute multiple scores for each word:

• : This scoring function, called *SlnSES*, enables the inclusion of sequence coverage into the score. A highly scored word occurs in a large percentage of sequences in the data set. It does not necessarily have to be highly significant if the overall number of occurrences is taken into account, but it is of particular use for the discovery of shared regulatory elements across multiple sequences.

• *p*-Value: The p-value is defined as the probability of obtaining at least as many words as the actual observed number of words: , where |*S*| represents the number of sequences in *S *and *l*_*j *_is the length of sequence *j*.

### Word-based clusters (SIG2)

Two methods are employed for the detection of similarities between the words that make up SIG1: hamming distance and Levenshtein distance (also called edit distance). While hamming distance is defined as the number of positions for which the corresponding characters of two words of the same length differ, edit distance allows the comparison of different length words and accounts for three edit operations (insert, delete and substitute), rather than the plain mismatch (corresponds to substitute) employed by the hamming distance.

The biological reasoning for employing distance metrics in order to group similar words together can be found in the evolution of sequences. A biological structure is constantly exposed to mutation pressure. These mutations can occur as insertions, deletions or substitutions, however insertions and deletions are deleterious in most cases, leading to the issue that edit distance provides a very detailed model of the mutations but hamming distance is a reasonable abstraction and will work well for this case. The motif logos for the hamming distance clusters were constructed using the TFBS Perl module by Lenhard and Wasserman [44]. ClustalW2 [45] was used to align the words of the edit distance clusters.

### Sequence clustering

The sequence clustering conducted in this research is focussed on the words shared between element of a set of sequences. A set of words is taken as the input for the clustering. A binary vector *s*_*i *_= (*s*_*i*,1_, *s*_*i*,2_,..., *s*_*i*, *k*_) for each sequence *s*_*i *_is created, marking an element *s*_*i*, *k *_where *k *is the number of words used to distinguish the sequences with *k *≤ |*W*^*wc*^|. The element *s*_*i*, *k *_of the vector is populated with a '1' if the word *k *is found in sequence *i*, and '0' if it is not. The similarity between sequences is determined by the dot product between the binary sequence vectors, and is deducted from the complete number of words in the vector space. In order to determine the distance between *k *sequences (with *k *≥ 2), the dot product is extended to accommodate multiple sequences.



The cluster with the smallest distance is visualized using GMOD's GBrowse framework [34]. For each of the sequences contained in the cluster, the words pertaining to SIG1 are displayed.

### Biological significance (lookup)

Once genomic signatures are identified, the next step is to discern their biological role. One important aspect of this role, crucial to understanding gene regulation [46], is the location of the preferred binding sites for certain proteins (transcription factor binding sites or TFBSs). To locate these sites, the signatures are compared to a set of known binding sites, which are usually represented as weighted matrices [47]. However, a simple scoring scheme can misclassify results when applied to the typically short sequences produced by signature finders. In this simple approach, short signatures are aligned to each matrix by ignoring the parts of the matrices that are longer than the signature. This results in erroneous scores since a signature could match just the very end of large matrix, which is often of little significance (the core of the matrix generally represents the sites of strongest binding).

To give a more significant measure of similarity, we developed a tool that uses a window around the original sequences (those which the signature is based upon) to improve the comparison. The naive implementation of this approach is to use a window of base pairs around each signature and find the optimal alignment to each TFBS matrix by scoring every possible sub-sequence containing the signature. For instance, if a signature is located 10 times within the set of sequences, each matrix is aligned to each of the 10 loci containing the signatures. Our tool uses a faster approach; it finds all occurrences of TFBSs meeting the desired threshold in every sequence, and subsequently uses this information to quickly score the signatures. As a benefit, the list of TFBS can be reused to quickly score new signatures or to redo the analysis with interesting subsets of sequences, such as all sequences which in liver cells are highly expressed.

### Co-occurrence analysis

The co-occurrence analysis aims to determine the expected number of sequences containing a given pair of not necessarily distinct words at least once. If *n *denotes the word length, *m *the number of sequences,  the probability for a word *i *to occur anywhere in the sequence, and *l*_*k *_the length of sequence *k*, the expected number of sequences containing a given pair of words can be calculated as:



The  score is used as the main scoring function in the co-occurrence analysis.

### Conservation analysis

Sequence conservation was mapped using PhastCons conservation scores [38] calculated on 28 species, which are based on a two-state (conserved state vs. Non-conserved region) phylo-HMM. PhastCons scores were obtained from the UCSC Human Genome Browser [39]. The scores reported by the UCSC Human Genome Browser contain transformed log-odds scores, ranging from 0–1000. Conserved regions were required to cover the majority of the word length.

### Comparison

Words can have significantly different scores for each of the data sets in which they occur. In order to analyze the words based on their impact on the data sets it is useful to assign a distance metric that determines which data set is described best by a given word. Based on a graphical analysis, three points of interest can be determined: the point where the perpendicular of a given point on the x-axis crosses the main diagonal, the point where the perpendicular of a given point on the main diagonal crosses the main diagonal and finally the point where the perpendicular from a given point on the y-axis crosses the main diagonal. Based on the conventional techniques of fold-change detection in microarray analysis, we consider the perpendicular on the main diagonal. The resulting distance formula is: , with *y*_0 _being the score for the word within the unidirectional data set, and *x*_0 _being the score of the word in the bidirectional data set.

## Competing interests

The authors declare that they have no competing interests.

## Authors' contributions

JL contributed in the development of algorithms and models, the implementation of algorithms, generation of the signature data and drafting of the document. EJ contributed the lookup of biological significance for the words of the signatures. JDW contributed in the development of the models and algorithms and the implementation of the approaches. KK contributed in the development and implementation of models and algorithms. XL contributed in the development of the models and algorithms for co-occurrence analysis and generated the respective data. MQY and LE generated and categorized the promoter data set. FD contributed in the development of models and algorithms, and in the implementation of the methods. KE contributed the idea of hamming-distance-based clustering. SSL contributed to the statistical foundations of the scoring model. LE provided the text describing the biological background and significance, conducted the conservation analysis, and participated in the drafting of the paper. In addition to architecting the software pipeline employed in this research, LRW contributed to the design, implementation and validation of models and algorithms (especially in the areas of word searching, word scoring, and sequence clustering) and to the writing of this manuscript.

## Supplementary Material

Additional file 1 **Words discovered in bidirectional promoters**. Entire set of words discovered in the bidirectional promoters with occurrences, expected occurrences, scores, reverse complement information and p-value. Click here for file

Additional file 2 **Words discovered in unidirectional promoters**. Entire set of words discovered in the unidirectional promoters with occurrences, expected occurrences, scores, reverse complement information and p-value. Click here for file

Additional file 3 **Missing words in bidirectional promoters**. Set of words not detected in the bidirectional promoters with expected occurrences. Click here for file

Additional file 4 **Missing words in unidirectional promoters**. Set of words not detected in the unidirectional promoters with expected occurrences. Click here for file

Additional file 5 **Hamming distance clusters in bidirectional promoters**. Entire set of hamming distance based clusters for the bidirectional promoters with detailed cluster element information position weight matrix and corresponding regular expression motif. Click here for file

Additional file 6 **Hamming distance clusters in unidirectional promoters**. Entire set of hamming distance based clusters for the unidirectional promoters with detailed cluster element information position weight matrix and corresponding regular expression motif. Click here for file

Additional file 7 **Edit distance clusters in bidirectional promoters**. Entire set of edit distance based clusters for the bidirectional promoters with detailed cluster element information position weight matrix and corresponding regular expression motif. Click here for file

Additional file 8 **Edit distance clusters in unidirectional promoters**. Entire set of edit distance based clusters for the unidirectional promoters with detailed cluster element information position weight matrix and corresponding regular expression motif. Click here for file

## References

[B1] Deschavanne PJ, Giron A, Vilain J, Fagot G, Fertil B (1999). Genomic signature: characterization and classification of species assessed by chaos game representation of sequences. Mol Biol Evol.

[B2] Karlin S, Burge C (1995). Dinucleotide relative abundance extremes: a genomic signature. Trends Genet.

[B3] Abe T, Kanaya S, Kinouchi M, Ichiba Y, Kozuki T, Ikemura T (2002). A novel bioinformatic strategy for unveiling hidden genome signatures of eukaryotes: self-organizing map of oligonucleotide frequency. Genome Inform.

[B4] Abe T, Kanaya S, Kinouchi M, Ichiba Y, Kozuki T, Ikemura T (2003). Informatics for unveiling hidden genome signatures. Genome Res.

[B5] Bastien O, Lespinats S, Roy S, Metayer K, Fertil B, Codani J, Marechal E (2004). Analysis of the compositional biases in Plasmodium falciparum genome and proteome using Arabidopsis thaliana as a reference. Gene.

[B6] Bentley SD, Parkhill J (2004). Comparative genomic structure of prokaryotes. Annu Rev Genet.

[B7] Campbell AM, Mrazek J, Karlin S (1999). Genome signature comparisons among prokaryote, plasmid, and mitochondrial DNA. Proc Natl Acad Sci.

[B8] Carbone A, Kepes F, Zinovyev A (2005). Codon bias signatures, organization of microorganisms in codon space, and lifestyle. Mol Biol Evol.

[B9] Deschavanne PJ, Giron A, Vilain J, Dufraigne C, Fertil B (2000). Genomic signature is preserved in short DNA fragments. IEEE International Symposium on Bioinformatics and Biomedical Engineering.

[B10] Elhai J (2001). Determination of bias in the relative abundance of oligonucleotides in DNA sequences. J Comput Biol.

[B11] Fertil B, Massin M, Lespinats S, Devic C, Dumee P, Giron A (2005). GENSTYLE: exploration and analysis of DNA sequences with genomic signature. Nucleic Acids Res.

[B12] Gentles AJ, Karlin S (2001). Genome-scale compositional comparisons in eukaryotes. Genome Res.

[B13] Jeffrey H (1990). Chaos game representation of gene structure. Nucleic Acids Res.

[B14] Karlin S (1998). Global dinucleotide signatures and analysis of genomic heterogeneity. Curr Opin Microbiol.

[B15] Karlin S, Campbell AM, Mrazek J (1998). Comparative DNA analysis across diverse genomes. Annu Rev Genet.

[B16] Karlin S, Mrazek J, Campbell AM (1997). Compositional biases of bacterial genomes and evolutionary implications. J Bacteriol.

[B17] Karlin S, Mrazek J, Gentles AJ (2003). Genome comparisons and analysis. Curr Opin Struct Biol.

[B18] Li J, Sayood K A Genome Signature Based on Markov Modeling. Proceedings of the 27th Annual International Conference of the IEEE – Engineering in Medicine and Biology Society: 2005; Shanghai.

[B19] Wong K, Finan TM, Golding GB (2002). Dinucleotide compositional analysis of Sinorhizobium meliloti using the genome signature: distinguishing chromosomes and plasmids. Funct Integr Genomics.

[B20] Zhang C, Zhang R, Ou H (2003). The Z curve database: a graphic representation of genome sequences. Bioinformatics.

[B21] Fickett JW, Wasserman WW (2000). Discovery and modeling of transcriptional regulatory regions. Curr Opin Biotechnol.

[B22] Schones DE, Sumazin P, Zhang MQ (2005). Similarity of position frequency matrices for transcription factor binding sites. Bioinformatics.

[B23] Wasserman WW, Fickett JW (1998). Identification of regulatory regions which confer muscle-specific gene expression. J Mol Biol.

[B24] Wasserman WW, Palumbo M, Thompson W, Fickett JW, Lawrence CE (2000). Human-mouse genome comparisons to locate regulatory sites. Nat Genet.

[B25] Joshi-Tope G, Gillespie M, Vastrik I, D'Eustachio P, Schmidt E (2005). Reactome: a knowledgebase of biological pathways. Nucleic Acids Res.

[B26] Yang MQ, Elnitski L, Zhang Y Heidelberg (2007). A computational study of bidirectional promoters in the human genome. Springer Verlag Lecture Notes in Bioinformatics.

[B27] Adachi N, Lieber MR (2002). Bidirectional gene organization: a common architectural feature of the human genome. Cell.

[B28] Yang MQ, Koehly LM, Elnitski LL (2007). Comprehensive annotation of bidirectional promoters identifies co-regulation among breast and ovarian cancer genes. PLoS Comput Biol.

[B29] Seila AC, Calabrese JM, Levine SS, Yeo GW, Rahl PB, Flynn RA, Young RA, Sharp PA (2008). Divergent Transcription from Active Promoters. Science.

[B30] Wingender E, Chen X, Hehl R, Karas H, Liebich I (2000). TRANSFAC: an integrated system for gene expression regulation. Nucleic Acids Res.

[B31] Bryne JC, Valen E, Tang MH, Marstrand T, Winther O (2008). JASPAR, the open access database of transcription factor-binding profiles: new content and tools in the 2008 update. Nucleic Acids Res.

[B32] Herold J, Kurtz S, Giegerich R (2008). Efficient computation of absent words in genomic sequences. BMC Bioinformatics.

[B33] Hampikian G, Andersen T (2007). Absent sequences: nullomers and primes. Pac Symp Biocomput.

[B34] Stein LD, Mungall C, Shu S, Caudy M, Mangone M, Day A, Nickerson E, Stajich JE, Harris TW, Arva A (2002). The generic genome browser: a building block for a model organism system database. Genome Res.

[B35] Jacox E, Elnitski L (2008). Finding Occurrences of Relevant Functional Elements in Genomic Signatures. International Journal of Computational Science.

[B36] Xie X, Lu J, Kulbokas EJ, Golub TR, Mootha V (2005). Systematic discovery of regulatory motifs in human promoters and 3' UTRs by comparison of several mammals. Nature.

[B37] Chekmenev DS, Haid C, Kel AE (2005). P-Match: transcription factor binding site search by combining patterns and weight matrices. Nucleic Acids Res.

[B38] Siepel A, Bejerano G, Pedersen JS, Hinrichs AS, Hou M, Rosenbloom K, Clawson H, Spieth J, Hillier LW, Richards S (2005). Evolutionarily conserved elements in vertebrate, insect, worm, and yeast genomes. Genome Res.

[B39] Kent WJ, Sugnet CW, Furey TS, Roskin KM, Pringle TH, Zahler AM, Haussler aD (2002). The Human Genome Browser at UCSC. Genome Res.

[B40] Bailey TL, Williams N, Misleh C, Li WW (2006). MEME: discovering and analyzing DNA and protein sequence motifs. Nucleic Acids Res.

[B41] Roth FP, Hughes JD, Estep PW, Church GM (1998). Finding DNA regulatory motifs within unaligned noncoding sequences clustered by whole-genome mRNA quantitation. Nat Biotech.

[B42] Lichtenberg J, Jacox E, Yang MQ, Elnitski L, Welch L (2008). Word-based characterization of the bidirectional promoters from the human DNA-repair pathways. The 2008 International Conference on Bioinformatics and Computational Biology.

[B43] Lichtenberg J, Morris P, Ecker K, Welch L (2008). Discovery of regulatory elements in oomycete orthologs. The 2008 International Conference on Bioinformatics and Computational Biology.

[B44] Lenhard B, Wasserman WW (2002). TFBS: Computational framework for transcription factor binding site analysis. Bioinformatics.

[B45] Larkin MA, Blackshields G, Brown NP, Chenna R, McGettigan PA, McWilliam H, Valentin F, Wallace IM, Wilm A, Lopez R (2007). ClustalW and ClustalX version 2.0. Bioinformatics.

[B46] Birney E, Stamatoyannopoulos JA, Dutta A, Guigo R, Gingeras TR, Margulies EH, Weng Z, Snyder M, Dermitzakis ET, Thurman RE (2007). Identification and analysis of functional elements in 1% of the human genome by the ENCODE pilot project. Nature.

[B47] Wasserman WW, Sandelin A (2004). Applied bioinformatics for the identification of regulatory elements. Nat Rev Genet.

